# An evaluation of clinical and ultrasound results of Pavlik harness treatment for developmental dysplasia of the hip

**DOI:** 10.25122/jml-2021-0289

**Published:** 2022-06

**Authors:** Seyed Rohallah Mousavibaygei, Amir Karimnia, Mohamad Hadi Gerami, Farhad Azadmehr, Taher Erfanifam, Amin Ghaedi

**Affiliations:** 1 Department of Surgery, School of Medicine, Qom University of Medical Sciences, Qom, Iran; 2 Department of Orthopedic Surgery, Imam Reza Hospital, Urmia University of Medical Sciences, Urmia, Iran; 3 Department of Orthopaedics, Bone and Joint Disease Research Center, Shiraz University of Medical Sciences, Shiraz, Iran; 4 Department of Medical Surgical Nursing, Boukan Nursing Faculty, Urmia University of Medical Sciences, Urmia, Iran; 5 Department of Surgery, Iran University of Medical Science, Tehran, Iran; 6 Department of Orthopaedics and Traumatology, Faculty of Medicine, Ahvaz Jundishapur University of Medical Sciences, Ahvaz, Iran

**Keywords:** hip dysplasia, Pavlik Harness treatment, hip dislocation

## Abstract

Developmental dysplasia of the hip (DDH) is the instability or dislocation of the hip joint at birth that may occur in utero, during infancy, and childhood. This condition was identified as an important challenge. This study aimed to determine the clinical and ultrasound results of Pavlik harness treatment for DDH in patients referred to the pediatric clinic of Imam Khomeini Hospital in Ahvaz. This is a descriptive cross-sectional study in which 100 newborns aged 15 to 30 days were included by the census method after obtaining parental consent. Follow-up of the infants was performed at 3 and 6 months after treatment. All analyzes were performed using SPSS version 22 at a significance level of 0.05. The results showed that the mean age of the infants was 23.46±2.12 days, of which 33 infants were boys and 67 girls. The involvement on the right and left sides was 39% and 50%, respectively, and 11% of the infants had bilateral involvement. The mean value of acetabular index before placement was 25.48±6.509 and 26.38±3.866 on the right and left sides, which after 3 months of placement, was reduced to 21.62±2.578 and 21.57±2.839, respectively. Pavlik harness treatment was associated with acceptable radiological results in infants. This technique seems to be a suitable and applicable attempt to treat this problem and prevent serious and irreversible complications of late diagnosis.

## INTRODUCTION

Developmental dysplasia of the hip (DDH) is the instability or dislocation of the hip joint at birth and a spectrum of developmental abnormalities of the hip that may occur during the embryonic period, infancy, or childhood [1]. This condition has long been an important challenge for pediatric orthopedics [2]. Since the first detailed diagnostic and therapeutic study by Ortolani, significant progress has been made in diagnosing, treating, and understanding the etiology of the disease [3]. Although the prevalence of the disease is reported to be one in 1,000 children, according to Barlow's classic study, the incidence is one case per 60 live births, of which, in 60%, the hip stabilizes within a week and in 88% within 2 months [4]. It is also more prevalent in some races, such as Native Americans and northern Finland (20 to 50 per 1000), and less common in some races, including South China and Africa [5].

The clinical course of DDH is often as full dislocation, limb shortening, lameness, pain, spinal osteoarthritis, and stunting in the patients' fifth decade of life [6]. It mainly appears as a shallow and superficial acetabulum, partial or full dislocation [7]. Although the main causes of DDH remain unknown, some of the factors contributing to DDH include gender, birth order, family history, intrauterine position, type of delivery, joint weakness, and postpartum positioning [8, 9]. Genetic factors are also involved in the development of this, so the involvement of one parent increases the risk of involvement in children by 10 times. 80% of infants with this complication are girls, and the risk of a breech position in these patients (about 20%) is higher than in the normal population (2–4%). Swaddling infants increases the risk of DDH [10]. The incidence of this disease is also associated with an increased risk of oligohydramnios and neuromuscular disorders [11]. This disease is generally more common in the left hip than in the right [12].

DDH causes the hip joint to grow abnormally and babies to have a relative ligament laxity at birth. Due to shallower acetabular cavities in white people, joint instability is more common [13]. This is one of the most common causes of hip reduction in patients under 60 years of age [14]. Treatment varies based on age and extent of dysplasia, and the prevalence of this disorder has been reported to be three times higher on the left side [15]. The preferred treatment for patients under three months of age is Pavlik Harness or dynamic hip abduction orthosis, while for patients 3 to 18 months (with or without atrophy), open and closed placement. Pelvic osteoid is used for patients 18 months and older [16].

Pavlik Harness is a soft brace usually used throughout the day for three months; the brace may be used part-time after recovery of the hip joint. Treatment with Pavlik Harness braces is successful in about 85% of dislocated hips in children under six months [17].

Ultrasound also plays an important role in diagnosing and treating this disease and is useful in monitoring closed reduction techniques [18]. Late diagnosis of DDH can lead to lameness and walking on the toes [19]. Early diagnosis and non-surgical treatment are important in reducing the risk of vascular necrosis, time of hospitalization, osteoarthritis, and treatment costs [20].

According to the above, clinical skills in the physical examination are essential in the early diagnosis of the disease, and radiography in suspected cases is a confirmatory tool for anomalies [21]. However, clinical symptoms are not always clear, and it is possible that the disease is diagnosed too late and results in serious and irreversible complications. In response to the diagnostic failures presented in clinical and radiological methods, the ultrasound method was introduced to screen and diagnose neonates [22]. The most comprehensive initial survey was performed using a graph by calculating alpha and beta angles and evaluating joint dynamics. Although false-positive results are common in ultrasound, currently, it is recognized as an accurate, safe, and effective tool in the early diagnosis of DDH [23]. Therefore, this study aimed to determine the clinical and ultrasound results of Pavlik Harness Treatment for DDH in patients referred to the pediatric clinic of Imam Khomeini Hospital in Ahvaz during 2019 to 2020.

## MATERIAL AND METHODS

This is a descriptive cross-sectional study conducted on a group of neonates with DDH treated with Pavlik harness referred to the pediatric clinic of Imam Khomeini Hospital in Ahvaz during 2019–2020. Inclusion criteria were willingness to participate in the study. Exclusion criteria were reluctance to participate in the study, neonates with connective tissue, secondary dislocation due to a previous infection, and acetabular dysplasia in a specific syndrome.

All neonates were selected and examined using the census sampling method (including the records of the infants admitted since 2019 and the infants referred after registering the code of ethics).

### Intervention

All infants underwent Barlow, Ortolani, telescoping, click and clunk tests followed by hip ultrasound. Ultrasound was applied in static and dynamic mode using a multi-frequency linear probe of 10MHz and an ultrasound device. In the static examination, the infants were placed at the supine position with legs in parallel, and an ultrasound with coronal incision was performed with the ultrasound probe at the outer margin of the hip joints. Specific angles α & β and sonographic type of the joints were determined. In the dynamic examination, the knees were in a flexed position, and the amount of displacement of the femoral head was recorded after applying posterior and lateral pressure on the femurs. Head displacement less than 6 mm was classified as the hypermobile femur, displacement greater than 6 mm as full dislocation as the head was completely out of the acetabular cavity, and brief displacement as a partial dislocation.

### Statistical analysis

Mean (and/or median) values were used to describe the data centers and standard deviations (and/or mid-quarter amplitude) to describe data scattering, while frequency and percentage were used to describe qualitative variables. When necessary, Chi-square (or Fisher's exact test) and t-test (or Mann-Whitney) were used to analyze the data. All analyzes were performed using SPSS version 22 at a significance level of 0.05.

## RESULTS

The demographic information of the patients is indicated in Table 1. The mean age of the infants was 23.46±2.12 days, of which 33 infants were boys and 67 girls. As shown in this table, the involvement on the right and left sides was 39% and 50%, respectively, and 11% of the infants had bilateral involvement.

**Table 1:** Distribution of patients' demographics in the two groups.

Variable		Frequency%
**Age (Mean±SD)**		**23.46±2.12**
**Gender (N, %)**	**Girl**	67 (67%)
	**Boy**	33 (33%)
**Involved side (N, %)**	**Right**	39 (39%)
	**Left**	50 (50%)
	**Bilateral**	11 (11%)

The evaluation of the acetabulum index based on clinical examination is shown in Table 2. According to these results, the mean value of acetabular index before placement was 25.48±6.509 on the right side and 26.38±3.866 on the left side, which after 6 months of placement, was reduced to 21.62±2.578 and 21.57±2.839, respectively, indicating an improvement in the acetabulum.

**Table 2:** Mean values of acetabulum index in primary and secondary (after 6 months) evaluation based on radiography results in the study group.

**Primary evaluation**	25.48±6.509	R	<0.001
	26.38±3.866	L	
**Secondary evaluation**	21.62±2.578	R	
	21.57±2.839	L	

The initial evaluation of the hip based on ultrasound, including α and β angles of the hip and their comparison after 3 months of the intervention, are shown in Table 3. The results showed that the angle α on the right and left sides increased from initial evaluation to 3 months after treatment, indicating an improvement and confirming the effect of treatment on the patient's recovery. On the other hand, the angle β reduced during this period, indicating an improvement in hip dislocation.

**Table 3:** Comparison of the change trends of α and β angles between right and left sides.

**Right α**	Primary	55.21±4.734	<0.001
	**After 3 months**	63.38±3.500	
**Right β**	**Primary**	54.14±8.737	<0.001
	**After 3 months**	46.76±11.58	
**Right α**	**Primary**	58.14±2.675	<0.001
	**After 3 months**	64.05±4.83	
**Right β**	**Primary**	57.81±7.033	<0.001
	**After 3 months**	46.76±11.554	

## DISCUSSION

Developmental dysplasia of the hip (DDH) includes a wide spectrum of disorders that arise from abnormal hip development that can occur at any time, including the embryonic period, infancy, or childhood. The main purpose of this descriptive-analytical study was to evaluate the clinical and ultrasound results of Pavlik harness treatment for infants suffering from DDH. In this study, the treatment records of 100 patients with DDH (including 67 girls (67%) and 33 boys (33%)) treated by Pavlik harness were evaluated.

Anari et al. (2007) conducted a study of sonographic and radiographic results in 100 infants (48 girls and 52 boys) with clinical findings of dislocated hips. The results showed that the positive findings of congenital hip dislocation in radiology and sonography were 28% and 56%, respectively [24]. The mean value of the initial acetabular index was 25.48±6.509 on the right side and 26.38±3.866 on the left side. After 6 months of surgery, these values decreased to 21.62±2.578 and 21.57±2.839, respectively. The results of this study are consistent with the findings of Sankar et al. (2019), who reported that the mean values of the acetabular index of patients decreased one year after treatment [20].

Sadoni et al. (2019) studied 30 children (33 joints) with DDH admitted to Razi Hospital from Ahvaz between 1994 and 1996. The results showed that the mean value of the post-surgery acetabular index (27.06±2.15) was significantly reduced compared to the preoperative value (36.54±3.27). The present study supports the results of the above study [25].

Based on the present study, a 6-month follow-up of patients treated with Pavlik harness showed positive effects and improvement in the children's condition, and clinical improvement was reported in the patients compared to preoperatively.

In this regard, Wahlen et al. (2015) stated that Pavlik harness treatment for DDH can be complex for parents. Any improper use or incorrect adjustments can lead to significant complications. The results showed a reduction in pelvic necrosis in 28 of 33 patients (85%) and avascular necrosis of the femoral head. Patients were followed up for at least one year and at most 4 years [26].

In their study, Ömeroglu et al. (2018) evaluated the effectiveness of Pavlik harness in treating DDH in children under six months of age and concluded that the significant differences in clinical and radiological results, the rate of failures and complications as well as ineffective variables on the treatment outcome are due to heterogeneity of the data and different methods used in various studies. The overall success rate of the treatment in the short, medium, or long term varies from 45% to 100% and is usually more than 75%. Moreover, the rate of osteonecrosis of the femoral head varies from 1% to 30%. Age, gender, radiological or clinical severity of initial hip pathology, and parental compliance are the determinants of treatment failure [27]. The present study agrees with the studies mentioned above, suggesting the ability of Pavlik harness treatment to improve the clinical condition of patients with DDH.

The results of a recent study showed that developmental dysplasia of the hip is prevalent, affecting 1% to 3% of all infants. One of the well-recognized phenomena in DDH is that early diagnosis and appropriate treatment during the first weeks of life promotes healthy hip development [28]. Dynamic splints are are a valuable therapeutic option for instability and dislocation, particularly if applied within 4–5 months. In addition, dynamic splints are poorly contraindicated. Static orthotics are also an efficient option, but only for stable hips or residual acetabular dysplasia [29].

On the other hand, Carlile et al. (2014) conducted a study to evaluate the hip condition using anterior ultrasound scanning during Pavlik harness treatment of HHD and reported different results from those obtained in the present study. In this study, 118 patients underwent ultrasound scanning, of which 103 patients were successfully treated. In children who received ultrasound scanning, treatment duration was reduced (p=0.011), and no difference was observed in the success of treatment (p=0.211) [30]. The discrepancy between the results of the studies may be due to differences in sample size, study duration, and different races and populations. One of the limitations of this study was the difficulty in following up with patients after discharge for several months.

## CONCLUSION

Pavlik Harness treatment was associated with acceptable radiological results in infants. This technique seems to be a suitable and applicable attempt to treat this problem and prevent serious and irreversible complications of late diagnosis.
